# Pneumopericardium with massive pericardial effusion in the setting of tuberculosis constrictive pericarditis: a rare case report

**DOI:** 10.1093/jscr/rjac380

**Published:** 2022-08-21

**Authors:** Said Abdirahman Ahmed, Mohamed Abdullah Mohamud, Yalçın Özkurt, Ishak Ahmed Abdi, Mohamed Omar Hassan

**Affiliations:** Cardiology Department, Mogadishu Somali-Turkish Training and Research Hospital, Mogadishu, Somalia; Cardiology Department, Mogadishu Somali-Turkish Training and Research Hospital, Mogadishu, Somalia; Cardiology Department, Mogadishu Somali-Turkish Training and Research Hospital, Mogadishu, Somalia; Cardiology Department, Mogadishu Somali-Turkish Training and Research Hospital, Mogadishu, Somalia; Cardiology Department, Mogadishu Somali-Turkish Training and Research Hospital, Mogadishu, Somalia

## Abstract

Constrictive pericarditis is an uncommon complication of acute pericarditis, mainly caused by non-idiopathic sources. Pneumopericardium is the presence of air in the pericardial sac resulting from various procedures and circumstances, including trauma, iatrogenic, non-iatrogenic and natural causes. Here, we report a 16-year-old girl who came to the cardiology outpatient complaining of weakness, abdominal distention and shortness of breath while lying down and exertion. An echocardiography evaluation revealed a thickened precordium and massive pericardial effusion. Pericardiocentesis was performed for diagnosis and treatment purposes. Despite the patient’s remaining symptomatic and having no improvement following the procedure, we decided to perform chest computed tomography, which revealed a thickened pericardium with pneumopericardium. Partial pericardiectomy was performed successfully, and the sample was sent to the pathology department, which confirmed tuberculous constrictive pericarditis. The patient’s symptoms improved, and she was discharged on postoperative Day 5 standing on her foot.

## INTRODUCTION

Pneumopericardium is the presence of air in the pericardial sac resulting from various procedures and circumstances, including trauma, iatrogenic, non-iatrogenic and natural causes [1].

Constrictive pericarditis is an uncommon and potentially fatal condition in which pericardial inflammation causes the heart to constrict, resulting in cardiac tamponade and right-sided heart failure [2].

Constrictive pericarditis is a rare condition that may be secondary to tuberculosis (TB), especially in developing nations, and remains the most prevalent cause of recurrent pericarditis [2, 3].

Here, we report a rare case of pneumopericardium with massive pericardial effusion in the setting of tuberculous constrictive pericarditis, which was managed successfully with partial pericardiectomy.

## CASE REPORT

A 16-year-old girl came to the cardiology outpatient complaining of weakness, abdominal distention and shortness of breath while lying down and exertion.

Physical examination revealed S1 + S2 + S4 + sinus tachycardia, 2+ pretibial edema, decreased breath sound, crepitation and abdominal distention.

**Figure 1:**
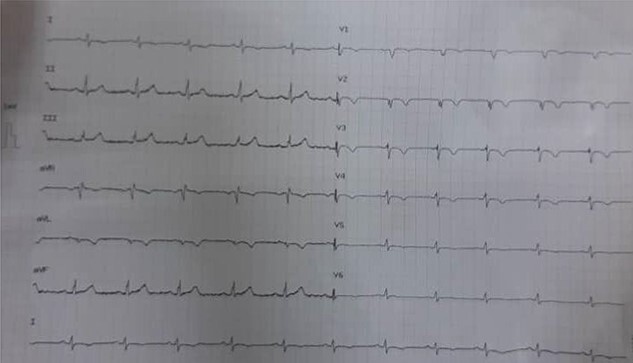
ECG shows low voltage.

The electrocardiography (ECG) revealed a low voltage with sinus tachycardia, indicating the existence of a pericardial effusion (Fig. 1).

**Figure 2:**
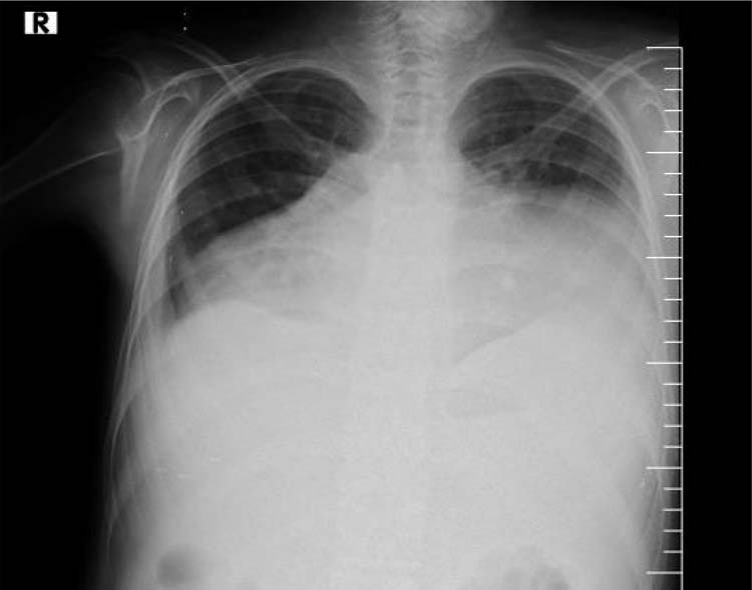
Chest X-rays show an enlarged cardiothoracic ratio, a lack of cardiac silhouette and pleural effusion.

**Figure 3:**
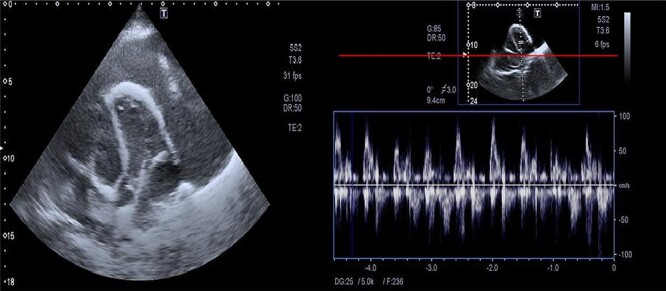
Massive precardial effusion with a swimming heart (**A**); respiratory variability at the mitral inflow level (**B**).

The assessment of the posterior anterior chest X-ray revealed an increased cardiothoracic ratio, a missing heart silhouette and pulmonary edema (Fig. 2). Furthermore, echocardiography evaluation revealed thickened pericardium, massive pericardial effusion, septal bounce and respiratory variation in mitral and tricuspid inflow (Fig. 3A and B).

Pericardiocentesis was performed for diagnosis and treatment purposes; during the procedure, 2 l of sero-hemorrhagic fluid were drained, and samples were sent to the microbiology unit.

**Figure 4:**
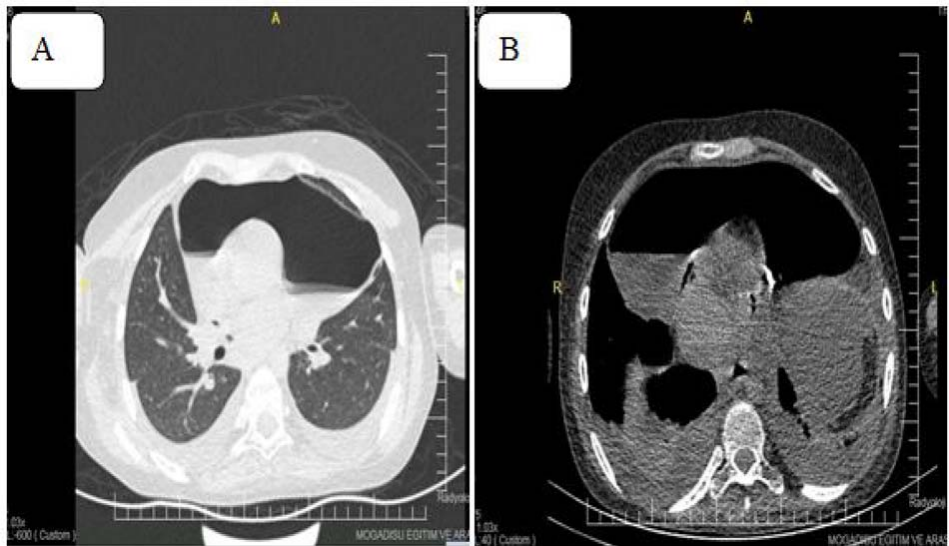
Pneumoprecadium on chest CT (**A**) and moderate pleural effusion (**B**).

Despite therapeutic Pericardiocentesis, the patient’s clinical has not resolved. Therefore, computed tomography (CT) of the chest was requested, which revealed a thickened pericardium with pneumopericardium, as shown in Fig. 4.

**Figure 5:**
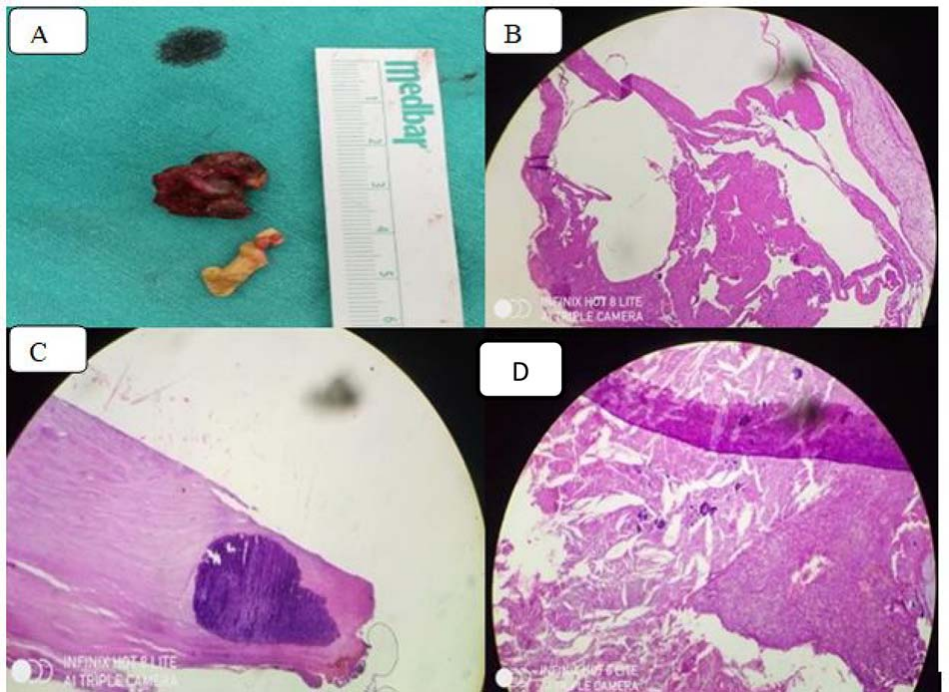
(**A**) Thickened pericardiumbiopsy; (**B**) chronic inflammation with granuloma formation on a fibrinous background (Hematoxylin and eosin stain); (**C**) diffuse calcification in the pericardium and (**D**) dystrophic calcification, macrophages, cholesterol clefts and chronic inflammation.

The patient was taken to the operation theatre, a partial pericardiectomy was performed successfully, and the sample was sent to the pathology department (Fig. 5). The entire procedure was uneventful. Although the sample of pericardiocentesis did not show any result of TB, the patient’s history of TB, echocardiography and thoracic CT images, and the sample of pathology support the diagnosis of constrictive pericarditis secondary to TB. Finally, postoperative echocardiography confirmed the success of the pericardiectomy, with the constrictive physiology seen before surgery resolved.

The patient’s symptoms improved, and she was discharged on postoperative Day 5, standing on her foot.

## DISCUSSION

Constrictive pericarditis is an uncommon complication of acute pericarditis, mainly caused by non-idiopathic sources. The presence of air in the pericardial cavity is referred to as pneumopericardium. It may occur due to pericardiocentesis or open-heart surgery, but it sometimes happens independently. Trauma from a penetrating or blunt chest injury is still the most prevalent cause [4]. Our case resulted from a pericardiocentesis procedure performed for diagnosis and treatment purposes.

TB is still the most prevalent cause of constrictive pericarditis in endemic areas like Somalia. This patient’s suspicion of tuberculous pericarditis was high because of his demographic risk factors and subacute clinical presentation [5].

Tuberculous constrictive pericarditis is characterized by pericardial constriction and diastolic dysfunction due to pericardial inflammation caused by *Mycobacterium tuberculosis* infection [6].

Distant heart sounds, a succussion splash with distended jugular veins, and shifting precordial tympany are common symptoms of tuberculous constrictive pericarditis. The present case presented with sinus tachycardia, reduced breath sound, crepitation and abdominal distention. Low voltage with sinus tachycardia was revealed by ECG [7].

All patients with heart failure should have an echocardiogram, which gives a valuable opportunity to check for constrictive pericarditis. The echocardiography’s diagnostic criteria for constrictive pericarditis are the ventricular septal shift, medial mitral e’ ≥ 9 cm/s, and hepatic vein expiratory diastolic reversal ratio of ≥0.79 [8]. Echocardiography of our case showed enlarged precordium, massive pericardial effusion, septal bounce and respiratory variability in mitral and tricuspid inflow.

The presence of air-fluid levels in the pericardium can be visualized via chest X-rays and CT scans [9]. Unstable patients with penetrating chest injuries and pneumopericardium necessitate emergent surgery [10]. The CT scan of our case revealed a thickened pericardium with pneumopericardium; similarly, an emergency pericardiectomy was performed, and the patient was transferred to the intensive care unit.

## CONCLUSION

Pneumopericardium caused by iatrogenic intervention can be treated conservatively and typically resolves without the need for surgery if timely diagnosed. Therefore, clinical and radiological evidence can be used to confirm a diagnosis of a tension pneumopericardium.

Our case demonstrates how early detection of a massive pneumopericardium can be life-saving.

## CONFLICT OF INTEREST STATEMENT

None declared.
